# Supplementary material for: Universal signatures of transposable element compartmentalization across eukaryotic genomes
Source: bioRxiv. 2024 Mar 27:2023.10.17.562820. Preprint. [Version 2] doi: 10.1101/2023.10.17.562820 (PMC10996525; doi:10.1101/2023.10.17.562820)
Supplement: Supplement 3 [file media-3.pdf]

Supplementary Table 1: Refseq (GCF) and Genbank (GCA) genomes considered in this study.

Supplementary Table 2: Final set of genomes after filtering.

Supplementary Table 3: GO enrichment results for TE-compartmentalized genes in each species. Only terms with Q<0.05 are displayed for species in which at least one term shows Q<0.05. For species where no terms achieve Q<0.05 enrichment, the top 5 most enriched terms with P<0.05 are shown. For full enrichment results, see Supplementary Data 1.

Supplementary Table 4: GO enrichment results for TE-compartmentalized genes across specific TE classes in each species. Only terms with Q<0.05 are displayed for species in which at least one term shows Q<0.05. For full enrichment results, see Supplementary Data 1.

Supplementary Table 5: Summary of enrichment results for terms shared across species.

Supplementary Table 6: Summary of enrichment results for terms shared across species for specific TE classes.

Supplementary Table 7: Multigene family enrichment results for TE-compartmentalized genes and other genes.

Supplementary Table 8: Multigene family enrichment results for TE-compartmentalized genes and other genes across specific TE classes.

Supplementary Table 9: Multidimensional scaling of significant (Q<0.05) Biological Process GO enrichment results aggregated across species.

Supplementary Table 10: Multidimensional scaling of significant (Q<0.05) Molecular Function GO enrichment results aggregated across species.

Supplementary Table 11: Multidimensional scaling of significant (Q<0.05) Cellular Component GO enrichment results aggregated across species.

Supplementary Table 12: Proportions of sites showing signatures of positive and negative selection for each TE-compartmentalized gene family and each other gene family in each species.

Supplementary Table 13: NCBI accessions for long-read *D. melanogater* assemblies used for population genetic analysis.

Supplementary Table 14: Comparison of enrichment results for LTRs and SINEs across all GO terms. For each term (column 1), FDR corrected Fisher's Exact P-values (column 2) are calculated by comparing the proportion of LTR-compartmentalized to the proportion of SINE-compartmentalized genes corresponding to that term.

Supplementary Table 15: Comparison of enrichment results for LTRs and LINEs across all GO terms. For each term (column 1), FDR corrected Fisher's Exact P-values (column 2) are calculated by comparing the proportion of LTR-compartmentalized to the proportion of LINE-compartmentalized genes corresponding to that term.

Supplementary Table 16: Comparison of enrichment results for LINEs and SINEs across all GO terms. For each term (column 1), FDR corrected Fisher's Exact P-values (column 2) are calculated by comparing the proportion of LINE-compartmentalized to the proportion of SINE-compartmentalized genes corresponding to that term.

Supplementary Table 17: GO enrichment results for TE-compartmentalized genes using a 50 kb flanking window size and 95th percentile repeat density threshold. Only terms with Q<0.05 are displayed for species in which at least one term shows Q<0.05. For species where no terms achieve Q<0.05 enrichment, the top 5 most enriched terms with P<0.05 are shown.

Supplementary Table 18: GO enrichment results for TE-compartmentalized genes using a 10 kb flanking window size and 90th percentile repeat density threshold. Only terms with Q<0.05 are displayed for species in which at least one term shows Q<0.05. For species where no terms achieve Q<0.05 enrichment, the top 5 most enriched terms with P<0.05 are shown.

Supplementary Table 19: GO enrichment results for TE-compartmentalized genes using a 10 kb flanking window size and 95th percentile repeat density threshold. Only terms with Q<0.05 are displayed for species in which at least one term shows Q<0.05. For species where no terms achieve Q<0.05 enrichment, the top 5 most enriched terms with P<0.05 are shown.

Supplementary Data 1: Raw GO enrichment results for TE-compartmentalized genes in each species, including genes driving enrichment for each GO term.

Supplementary Data 2: Clade-specific GO enrichment result summaries.
